# Effect of the soft tissue artifact on marker measurements and on the calculation of the helical axis of the knee during a gait cycle: A study on the CAMS-Knee data set

**DOI:** 10.1016/j.humov.2021.102866

**Published:** 2021-12

**Authors:** Andrea Ancillao, Erwin Aertbeliën, Joris De Schutter

**Affiliations:** a Robotics Research Group, Dept. of Mechanical Engineering, KU Leuven, 3001 Leuven, Belgium; b Flanders Make, Core Lab ROB, KU Leuven, 3001 Leuven, Belgium

**Keywords:** Fluoroscopy, Gait analysis, Helical axis, Knee prosthesis, Motion capture, Skin artifact, Soft tissue artifact

## Abstract

The soft tissue artifact (STA) is a phenomenon occurring when the motion of bones or anatomical segments is measured by means of skin markers: the biological tissues between the markers and the bone produce a relative motion bone-markers that leads to inaccuracies in the estimation of rigid body poses or kinematics. The aim of this study was to quantify the STA by exploiting a recently published gait analysis dataset. The dataset was composed of six adult subjects with a total knee arthroplasty who underwent gait analysis trials. The motion of the knee was concurrently recorded by means of (i) fluoroscopy imaging and (ii) an optoelectronic system and redundant markers attached to the thigh and shank. The STA was studied by comparing the results calculated on the marker sets with the results obtained from the fluoroscopy data. The stance and swing phases were considered separately. Rigid STA motion and local STA deformation were studied separately. In addition to previous studies, the instantaneous helical axis (IHA) of the knee was calculated and the effect of the STA on its calculation was assessed.

The largest rigid-motion STA effect was observed on the thigh cluster (~10 deg. and ~ 18 mm). The shank cluster was mainly affected during the swing phase (~7 deg. and ~ 17 mm). The local STA deformation affected differently the markers. The largest effect was ~16 mm and the lowest was ~4 mm. The estimation of the IHA was not reliable when based only on markers, having an estimation error of ~17 deg. and ~ 25 mm. A high variability of results across subjects was observed.

## List of Abbreviations

AHA: Average Helical Axis
IHA: Instantaneous Helical Axis
LCSF: Local Coordinate System defined by Fluoroscopy
LCSM: Local Coordinate System defined by Markers
LSTAD: Local Soft Tissue Artifact Deformation
PCT: Point Cluster Technique
RSTAM: Rigid Soft Tissue Artifact Motion
SD: Standard Deviation
STA: Soft Tissue Artifact

## 1 Introduction

In clinical contexts, the motor performance of patients is commonly recorded by means of optoelectronic systems that record the position of passive markers attached on the skin of the subjects (Ancillao, 2018). Gait analysis is a typical example of a motion analysis examination commonly conducted in the clinical routine (Baker et al., 2009). Such motion capture measurements are usually considered reliable for clinical purposes (Ancillao et al., 2017; Ferrari et al., 2008; Kadaba et al., 1989; Stief, Böhm, Michel, Schwirtz, & Döderlein, 2013). Most of the anatomical protocols adopted for the quantification of kinematics and kinetics assume the anatomical segments (e.g. the thigh or the shank) as rigid bodies (Ancillao, 2018; Davis, Ounpuu, & Gage, 1991). However it is well known that the marker measurements are affected by the soft tissue artifact (STA) (Leardini, Chiari, Croce, & Cappozzo, 2005). The STA represents the failure of the rigid body hypothesis due to the relative motion between the skin and the underlying layers (fat, muscles, bones) occurring when the subject moves and it may lead to a significant error on the estimation of the quantities of biomechanical interest, such as the joint kinematics.

In order to completely avoid STA, motion capture markers should be rigidly fixed to the underlying bone by means of screws (Blankevoort, Huiskes, & de Lange, 1990; Leardini et al., 2005), but this is a highly invasive procedure that cannot be adopted in the daily clinical routine. The non-invasive markers attached to the skin remain therefore the most accepted method to record the motion.

Several methods to reduce the effect of STA were proposed: the easiest one consists in the specific design of marker protocols having marker landmarks where the STA is expected to be the minimum. Examples of such points are the bone epicondyles that are easily palpable through the skin. Several examples of such protocol design can be found in the literature, such as the Davis protocol (Davis, R.B.; Ounpuu, S.; Gage 1991), the “IfB” Marker Set (List, Gülay, Stoop, Lorenzetti, & Renate, 2013), the “OSSCA” (Taylor et al., 2010). Alternatively, it was proposed to adopt technical protocols where marker clusters are rigidly attached to plates that are fixed to the skin (Cappozzo, Cappello, Croce, & Pensalfini, 1997; Cappozzo, Croce, Leardini, & Chiari, 2005). It should be remarked that these protocols were designed mainly for clinical use, where the time required to vest the subject plays an important role, and the effect on STA reduction is minimal (Garling et al., 2007). To further reduce the STA, several methods involving optimization procedures were proposed. These methods exploit redundant marker measurements in order to minimize the distance between their measured position and a reference configuration, typically a standing trial. Examples are the Point Cluster Technique (PCT) (Alexander & Andriacchi, 2001; Andriacchi, Alexander, Toney, Dyrby, & Sum, 1998) or the Optimal Common Shape Technique (Taylor et al., 2005). These methods are not typically exploited in the daily clinical procedures because of the longer preparation time and the requirement of advanced skills for the data processing that requires trained operators.

It was proven that the STA can be very different from subject to subject, it is not possible to build a unique model and, in general, removal of STA is not an easy task (Peters, Galna, Sangeux, Morris, & Baker, 2010), although it was suggested that the STA may be correlated to some physical characteristics of the subject, such as age, body-mass index, skin stiffness, fat, etc. (Garling et al., 2007; Peters et al., 2010), and to the specific motor task executed (Leardini et al., 2005). Several studies attempted to quantify the artifact due to the skin motion (Cappozzo, Catani, Leardini, Benedetti, & Della Croce, 1996; Manal, Davis, Galinat, & Stanhope, 2003; Reinschmidt et al., 1997), observing a relative skin to bone marker movements in the range of 3 mm up to 40 mm dependent upon the specific body segment (Taylor et al., 2005). Another study reported STA errors ranging from 1.6 deg. to 22.4 deg. for knee rotations and from 0.8 mm to 14.9 mm for knee displacements in healthy subjects (Clément, Dumas, Hagemeister, & de Guise, 2015). The same study remarked that the best way to attenuate the STA is by adopting subject-specific models, which is in practice not convenient for the daily clinical routine. A recent study suggested that STA affecting a body segment can be modelled according to two components: (i) the rigid body relative displacement of skin over the bone, namely “rigid STA movement”, RSTAM; and (ii) the skin surface deformation, namely “local STA deformation”, LSTAD (Barre, Thiran, Jolles, Theumann, & Aminian, 2013). It was observed that during a walk on a treadmill, the RSTAM amplitude represented the 80–100% of the total STA. The thigh segment was the most affected and most of the motion occurred along the vertical axis of the femur. It was remarked that barefoot walking over ground may produce different results with respect to the treadmill (Barre et al., 2013).

In general, previous studies showed that STA is dependent upon marker landmarks and activity performed. Even if STA is intra-subject repeatable, it was not repeatable across different subjects. In some subjects, some body segments may be more affected than others, depending on subjects' physical characteristics. Finally, the frequency components of the STA are comparable to the frequency of the motor task. Currently there are no conclusive methods to completely remove STA in human motion analysis (Peters et al., 2010).

The STA may have a strong impact on the calculation of functional quantities, such as the helical axis or instantaneous centre of rotation of a joint, that require the calculation of differential kinematics and strongly rely on accurate marker measurements (Ancillao, Vochten, Aertbeliën, Decré, & De Schutter, 2019; Ehrig, Taylor, Duda, & Heller, 2007; Ferraresi, De Benedictis, Franco, Maffiodo, & Leardini, 2017; Sancisi, Parenti-Castelli, Corazza, & Leardini, 2009). A previous study demonstrated that the Gaussian noise had an impact on the calculation of the helical axis and the effect was larger for the smaller angular displacements of the joint (Cescon, Cattrysse, & Barbero, 2014). Other works demonstrated that, even if the physiological knee motion has some known out-of-plane rotations (in the two planes other than the sagittal), the measurement of these rotations is not reliable when conducted by the common clinical protocol, due to the large artifact on the markers (Cutti et al., 2010; Ramsey & Wretenberg, 1999; Stagni, Fantozzi, & Cappello, 2006).

## 2 Study aim

The aim of this study was to quantify the effects of STA occurring on the lower limbs during a gait analysis trial. To this purpose, we exploited the CAMS-Knee dataset that includes several gait analysis trials concurrently recorded by means of redundant marker sets and fluoroscopy-based direct measurement of the poses of femur and tibia (Taylor et al., 2017).

The main scopes of this analysis were: (i) evaluate the effect of the STA on the calculation of the helical axis of the knee and (ii) quantify STA occurring on thigh and shank clusters by studying separately the effect of RSTAM and LSTAD and studying separately the stance phase and swing phase of the gait cycle.

## 3 Materials and methods

### 3.1 Dataset

This work took advantage of the CAMS-Knee dataset (Taylor et al., 2017), consisting in multifactorial gait analysis recordings of six subjects (5 m, 1f, aged 68 ± 5 years, mass 88 ± 12 kg, height 173 ± 4 cm) each with an instrumented total knee prosthesis (Schütz, Taylor, Postolka, Fucentese, & Koch, 2019). Four subjects had the implant on the left knee, two had the implant on the right knee. Whole body kinematics was recorded by means of an optoelectronic system and a total of 75 skin markers. Sampling frequency was 100 Hz. Motion of the instrumented knee implant was also dynamically tracked by means of a video-fluoroscope mounted on an automated trolley. Fluoroscopy images were corrected for distortion and the three-dimensional poses of femoral and tibial components of the implant were reconstructed with an estimated accuracy of <1 degree for all rotations, <1 mm for in-plane and < 3 mm for out-of-plane translations (Taylor et al., 2017).

### 3.2 Body segments

The present study focussed on the kinematics of the knee during the gait. Thus, the considered body segments where the thigh/femur and shank/tibia on the side of the implant. The motion of the thigh was tracked by means of 8 markers; the motion of the shank was tracked by means of 10 markers. The detailed marker names and the respective landmarks are listed in Table 1. Marker landmarks were chosen according to the Plug In Gait, IfB and OSSCA protocols (Taylor et al., 2017). The data from the fluoroscopy processing consisted in the time-series poses of femoral and tibial components that were assumed rigidly attached to the femur and tibia respectively.

**Table 1:** List of the markers used in the study and location of the respective landmarks.

Marker	Landmark
Thigh	
TLH	Lateral thigh on 50% thigh length
TLL	Lateral thigh on 20% thigh length
TLE	Lateral epicondyle
TME	Medial epicondyle
TFR	Front thigh, one hand above knee
TAT	Ventral thigh on 50% of the length
TPP	Upper 1/3 of the dorsal thigh
TPD	Lower 1/3 of the dorsal thigh

Shank	
THF	Head of fibula
TTT	Tibial tuberosity
TMT	Mid tibia on 50% shank length
TDT	Lower 1/3 of the ventral shank
TLF	Lateral fibula on 30% shank length
TLS	Upper 1/3 of the lateral shank
SPP	Upper 1/3 of the dorsal shank
SPD	Lower 1/3 of the dorsal shank
TLM	Lateral malleolus
TMM	Medial malleolus

### 3.3 Data pre-processing

The data included in the study were the marker trajectories and poses measured by fluoroscopy. The marker trajectories were low-pass filtered (10 Hz) for anti-aliasing, then resampled to match the sampling frequency of the fluoroscopy dataset i.e. 25 Hz and were further smoothed by using a recursive moving average filter (Ancillao, Galli, Vimercati, & Albertini, 2013; Ancillao, Savastano, Galli, & Albertini, 2017).

The measured markers were grouped into two clusters: thigh and shank that were assumed as representative of the respective body segments. Only thigh and shank clusters were considered in the present study because of the availability of fluoroscopy measurements on those segments.

**Fig. 1:**
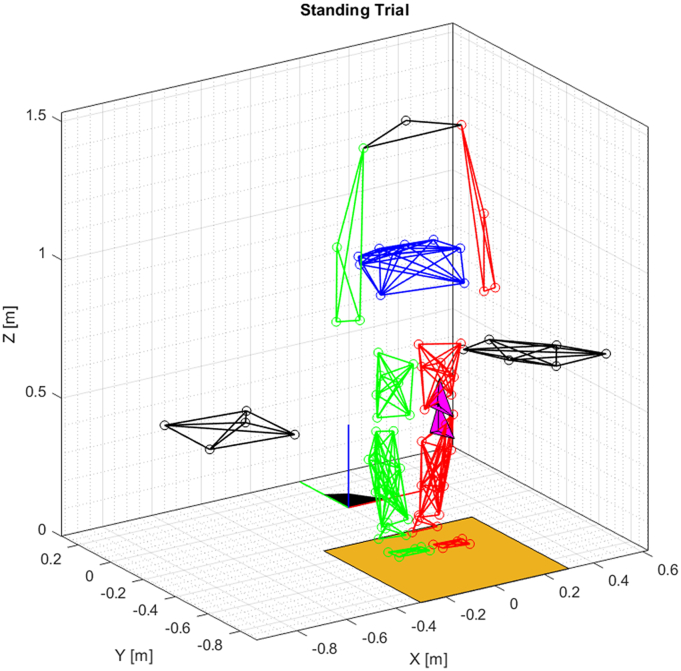
The full marker set during the standing trial, assumed as the reference configuration. The magenta tetrahedrons represent the poses of femur and tibia as recorded by the fluoroscopy. (For interpretation of the references to colour in this figure legend, the reader is referred to the web version of this article.)

For each time-frame, the marker-based pose of each segment and the optimized marker coordinates were reconstructed according to the PCT procedure, i.e. an optimization procedure that minimizes the distance between the measured coordinates and a reference configuration (Alexander & Andriacchi, 2001; Andriacchi et al., 1998). This procedure allows to define a coordinate system attached to the marker cluster in the reference configuration, and then optimally reconstruct the same coordinate system for each frame of the trial. For this study, we took the standing trial (Fig. 1) as the reference configuration, assuming that no STA was occurring during this trial. In the reference configuration, the marker-based local coordinate system (LCSM) was defined as coincident to the fluoroscopy-based local coordinate system (LCSF). The LCSF was directly provided by the data and it was defined as rigidly attached to the tibial and femoral components of the prosthesis (Taylor et al., 2017).

Finally, data were segmented according to gait cycles. The full gait cycle was defined as the data in-between the first heel-strike and the second heel-strike. The stance phase was defined as the data in-between the first heel-strike and the toe-off, the swing phase as the data between the toe-off and the second heel-strike. All the events were provided by the dataset. The segmented data were visually inspected in order to exclude cycles containing artefacts. For each subject, from 3 to 5 “good” cycles were included in the study. The data available for each gait cycle for both thigh and shank clusters were:

• LCSF
• LCSM
• Measured markers (meas.)
• PCT-optimized markers

**Fig. 2:**
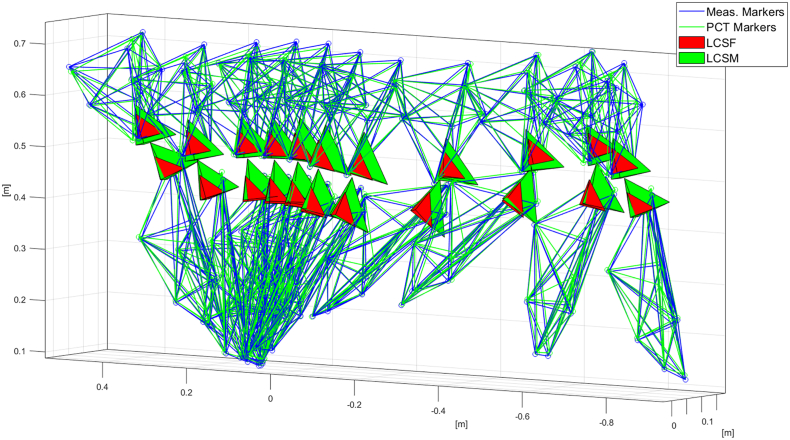
Example of a dataset used for STA analysis, relative to one gait cycle of one subject. The thigh and shank clusters are visualized. The tetrahedrons represent the coordinate systems attached to the clusters.

A three-dimensional visualization of the full marker set in the reference configuration is depicted in Fig. 1 while the marker sets and the LCSF and LCSM during a walking trial are depicted in Fig. 2. A more detailed figure depicting the full measured marker positions is available in (Taylor et al., 2017).

### 3.4 STA analysis

The STA was studied by comparing the motion of the body segments as reconstructed from the marker-based measurements to the fluoroscopy-based measurements, assumed as the gold standard. The analysis was divided into three parts: (i) analysis of the rigid body motion occurring between the skin and underlying bone for each body segment (RSTAM); (ii) quantification of the effect of skin deformation (LSTAD); (iii) analysis of the effect of STA on the calculation of the instantaneous helical axis of the knee (IHA).

To quantify the RSTAM, the homogeneous matrices representing the LCSM and the LCSF were compared. The two coordinate systems were depicted as the green and red tetrahedrons in Fig. 1. The comparison was done in terms of (i) translation and (ii) rotation, after decomposing the homogeneous matrices into the rotation part and origin. The translation was measured as the linear distance between the origins of LCSM and LCSF, while the rotation was quantified by means of the axis-angle representation of the relative rotation matrix between the two coordinate systems (Barre & Armand, 2014).

To quantify the LSTAD, the barycentre of each marker cluster was calculated and the Euclidean distance of each measured marker from the barycentre was calculated. The mean value of each distance was subtracted from the respective instantaneous distance in order to remove the constant offset and to quantify only the variation (displacement) of each marker with respect to the barycentre. In addition, the Euclidean distance between measured markers and PCT markers was computed to quantify the residuals of the PCT.

To quantify the STA effect on the IHA, the knee IHA, representing the relative motion of shank and thigh, was calculated two times: (i) based on the LCSF and (ii) based on the LCSM. The procedure for calculating the knee IHA was based on the relative screw twist between the shank and the thigh, according to previous literature (Ancillao et al., 2019; Schutter and Joris, 2010; Stokdijk, Meskers, Veeger, De Boer, & Rozing, 1999). The IHA was expressed in terms of its direction vector *n* and its position vector *S*:

(1) $$\omega =\sqrt{\omega^{T}∙\omega }$$

(2) $$n=\frac{\omega }{\omega }$$

(3) $$S=\frac{\omega \times v_{0}}{\omega^{2}}$$

where ***ω*** and ***v***_**0**_ are the components of the screw twist of the considered coordinate system.

The IHA is known to be ill-defined when *ω* is low. Thus the IHA was calculated only for *ω* ≥ 0.3 rad/s, as suggested by literature (De Rosario, Page, and Mata 2014; Stokdijk et al., 1999).

To analyse the effect of STA, the variation of the IHA with respect to a reference axis was calculated. The reference axis was functionally calculated as the average helical axis (AHA) according to the procedure described in (Ehrig & Heller, 2019; Stokdijk et al., 1999). More in details, the position of the AHA, P, was the pseudo-intersection of the IHAs, also named “optimal pivot point” that can be assumed as the center of rotation of the joint (Stokdijk et al., 1999). It could be obtained according to the following equations:

(4) $$Q_{i}=I-n_{i}{n_{i}}^{T}$$

(5) $$P={\left(\frac{1}{N}\sum_{i=1}^{N}Q_{i}\right)}^{-1}\left(\frac{1}{N}\sum_{i=1}^{N}Q_{i}S_{i}\right)$$

where ***n***_***i***_ is the direction of the i-th IHA and *I* is the 3 × 3 identity matrix. The direction *m* of the AHA, also named “optimal direction vector” was obtained from the following equation (Stokdijk et al., 1999):

(6) $$d={\left(\frac{1}{N}\sum_{i=1}^{N}Q_{i}\right)}^{-1}\left(\frac{1}{N}\sum_{i=1}^{N}Q_{i}n_{i}\right)$$

(7) $$m=\frac{d}{\left\|d\right\|}$$

The instantaneous deviation was calculated in terms of (i) angle and (ii) distance between IHA and AHA.

### 3.5 Data analysis and statistics

All the previously described parameters were calculated for all the gait cycles included in the study. To have a reasonable quantitative estimation of the STA effect, the range of variation for each parameter was calculated. The ranges were calculated separately for the stance phase and for the swing phase. In the case of the IHA, the deviation was calculated in terms of (i) angle and (ii) distance between IHA and AHA. Then, the range, the mean, and standard deviation across the whole gait cycle were calculated.

The results of each parameter were presented as mean values and standard deviations calculated across all the trials included in the study. The data groups were tested for normality by means of the Shapiro-Wilk test. As most of the data did not follow the normal distribution, the non-parametric Wilcoxon test was used to assess differences. The following comparisons were tested: (i) stance vs. swing and (ii) thigh vs. shank. For all the tests it was assumed α = 0.05.

All the data processing was done in MATLAB R2020b (www. mathworks.com).

## 4 Results

### 4.1 RSTAM

**Fig. 3:**
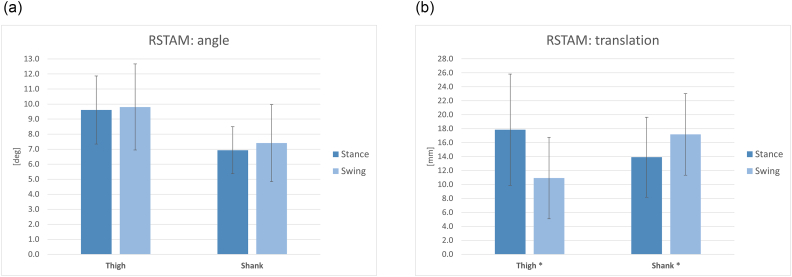
rigid STA motion between marker cluster and underlying bone, (a) total rotation angle, (b) translation., * significant differences stance-swing with *p* < 0.05.

The rigid STA motion was quantified in terms of angle (Fig. 3a) and translation (Fig. 3b). The RSTAM angle was always larger for the thigh, both in stance and swing. Significant differences (*p* < 0.05) were observed between stance and swing in the case of RSTAM translation. The translation was larger for the thigh during stance and larger for the shank during swing. The comparison between thigh and shank was always significant with *p* < 0.05.

During stance, the thigh was more affected by RSTAM, while, during swing, the translation was larger for the shank. The largest angular displacement was 10 ± 3 deg.; the largest translation was 18 ± 8 mm.

### 4.2 LSTAD

**Fig. 4:**
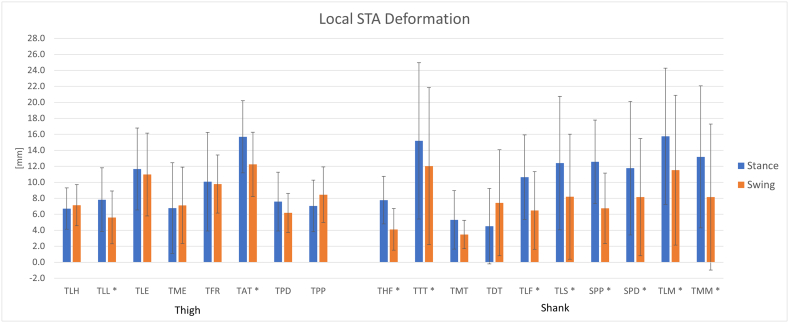
local STA deformation for thigh and shank during stance and swing phases of the gait cycle., * significant differences stance-swing with p < 0.05.

The local STA deformation was studied by means of the variation in distance with respect to the barycentre (Fig. 4). In general, the effect of LSTAD was larger during the stance phase. The largest displacement observed for the thigh was 16 ± 5 mm and for the shank was 16 ± 9 mm. The most affected markers where: TAT, TTT, TLM. In the case of the shank, higher variability of results across subjects was observed, as indicated by the larger SD (error bars in Fig. 4). In addition, the distance between the measured marker positions and the PCT optimized markers was measured. The largest residual for the thigh was 18 ± 6 mm, observed during the stance phase; and for the shank it was 17 ± 11 mm, observed during the swing phase. The significant differences were illustrated in Fig. 4. All the differences had *p* < 0.01;

### 4.3 IHA

**Fig. 5:**
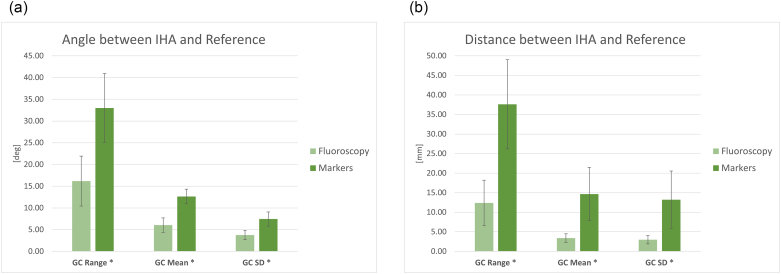
Deviation of the IHA from the reference, i.e. the AHA: (a) angular displacement and (b) linear translation. Mean, SD and range values with respect to the gait cycle (GC). * significant differences with p < 0.05.

The deviation of instantaneous helical axis of the knee from the reference axis is reported in Fig. 5a and b. After applying the threshold, the remaining samples were 38 ± 4% of gait cycle in the case of fluoroscopy and 39 ± 6% of gait cycle in case of markers. The IHA was instantaneously compared to the AHA, assumed as the reference and the variations calculated from the fluoroscopy data were compared to the ones based on the marker clusters. The IHA deviation was always statistically higher in the case of markers. The ranges of deviation were: 16 ± 6 deg. and 12 ± 6 mm in the case of fluoroscopy and 33 ± 8 deg. and 38 ± 11 mm in the case of markers. The mean deviation was 6 ± 2 deg. and 3 ± 1 mm for fluoroscopy and 13 ± 2 deg. and 15 ± 7 mm for markers. The SD across the gait cycle was 4 ± 1 deg. and 3 ± 1 mm for fluoroscopy and 8 ± 2 deg. and 13 ± 7 mm for markers. The SD across subjects/trials for translation was larger in the case of markers, suggesting an important dispersion of the results. All the differences were statistically significant with *p* < 0.01.

## 5 Discussion

In this study, the STA effect on marker measurements was analysed by comparison to the direct measurement of bone motion by means of fluoroscopy tracking, that was assumed as the gold standard. The fluoroscopy-based measurements were assumed as free of STA and their measurement accuracy was comparable to marker measurements (Taylor et al., 2017). The use of fluoroscopy imaging is considered the state of the art for accurately tracking the motion of bones or implants for in-vivo studies, in contrast to other methodologies such as simulations, models, ex-vivo samples or in-vivo intra-cortical pins (Peters et al., 2010). The main strengths of this study are: (i) the separate analysis of RSTAM and LSTAD; (ii) the separate analysis of swing and stance phases; (iii) the study of the effect of STA on the calculation of the IHA.

The observed RSTAM angle may significantly affect the measurements of small anatomical angles, such as the knee and ankle motion on the frontal and horizontal planes, as already reported in previous studies (Akbarshahi et al., 2010; Garling et al., 2007). Therefore, caution should be paid when interpreting such measurements obtained from skin-mounted markers. The swing phase increased the RSTAM on the shank, while during stance the largest effect was on the thigh.

Comparing the magnitude of RSTAM displacements to the LSTAD, it can be observed that RSTAM had a larger effect then LSTAD for most of the markers. Some markers showed a larger LSTAD, mainly during the stance phase. From these findings we can conclude that STA is governed by both RSTAM and LSTAD. For this reason, the procedures based on the solidification of the marker cluster, such as the PCT, may be not very effective in reducing the STA. In fact, this was confirmed by the maximum residuals observed for the case of PCT clusters that was ~18 mm comparable to the observed RSTAM displacement. The findings of the present study are coherent with previous studies that suggested an important effect of RSTAM especially on the thigh cluster, however it is well known that STA can have a very different pattern in different subjects (Akbarshahi et al., 2010; Barre et al., 2013; Leardini et al., 2005). With respect to the RSTAM and LSTAD mean values observed in this study, our findings are comparable to results reported in previous studies. For example, (Garling et al., 2007) reported a maximum translational error of 17 mm and a maximum rotational error of 12 deg.; (Andersen, Damsgaard, Rasmussen, Ramsey, & Benoit, 2012) reported a maximum residual due to STA of ~27 mm; for the thigh and ~ 24 mm for the shank; (Akbarshahi et al., 2010) reported a RMS value of ~19 mm; in some cases, an error up to 40 mm was observed (Peters et al., 2010).

The results about the calculation of the IHA are particularly interesting. The IHA represents the rotation axis of the joint as calculated in a functional way, and it plays an important role for the design of prostheses or for the analysis of the healthiness of the joint/ligaments (Ancillao, Rossi, & Cappa, 2017; Grip & Häger, 2013; Stokdijk et al., 1999). In this study we investigated the variation of the IHA in terms of angle and translation with respect to a reference axis that was calculated as the average helical axis according to an optimization procedure. A certain amount of IHA variation across the gait cycle, both in angle and translation, is physiological. In this study, we assumed the range measured by the fluoroscopy dataset as the physiological motion of the knee axis and the observed difference markers-fluoroscopy as the effect of STA. The ranges observed in the case of fluoroscopy (Fig. 5) are coherent with the physiology of the knee (Mannel, Marin, Claes, & Dürselen, 2004). The difference between marker and fluoroscopy is the effect of STA. From Fig. 5, the STA had an important effect on the calculation of the IHA, leading to a significant over-estimation of its variations both in terms of angles and distances. It is therefore not recommended to calculate the IHA of the knee based on markers.

The accuracy of the IHA calculation depends on the instantaneous magnitude of the angular velocity. For this reason, the IHA was not calculated for the gait cycle samples where the angular velocity magnitude was below the threshold. Increasing the threshold would increase the accuracy but it would leave out more samples, therefore less joint motion can be represented (Stokdijk et al., 1999). The threshold chosen in this work seemed a good compromise. The need of a threshold on the angular velocity or on the angular displacement is a known limitation of the IHA method (De Rosario, Helios, & Mata, 2014; Stokdijk et al., 1999), therefore the IHA results should always be interpreted with respect to the chosen threshold.

For all the parameters examined in the present study, a high variability across subjects was observed, as indicated by the relatively large standard deviation (see error bars in Fig. 3, Fig. 4, Fig. 5), this finding confirms the assumption that the effect of STA is subject-specific.

## 6 Conclusion

In this paper we studied the soft tissue artifact on marker measurements by comparison with the direct measurement of bone poses via fluoroscopy imaging. During a gait cycle, both RSTAM and LSTAD gave an important contribution to the STA. In general, it was observed that the thigh cluster was the most affected by STA and the effects were highly variable across the subjects. The findings of this study suggested that some attention should be paid when clinically relevant quantities are deducted from marker measurements. The measurement of small displacements or small rotations (angles) during gait is not reliable when based only on skin-attached markers. The largest errors due to STA were assessed as ~10 deg. and ~ 18 mm. Concerning the calculation of the IHA, it was found that it was not reliable when based on the marker measurements. The calculation of IHA based on markers led to an overestimation of the angular variation of ~17 deg. and overestimation of translational displacement of ~25 mm.

## Declaration of Competing Interest

The authors declare no conflict of interests.
